# High efficacy of the BCL-2 inhibitor ABT199 (venetoclax) in BCL-2 high-expressing neuroblastoma cell lines and xenografts and rational for combination with MCL-1 inhibition

**DOI:** 10.18632/oncotarget.8547

**Published:** 2016-04-01

**Authors:** Laurel T. Bate-Eya, Ilona J.M. den Hartog, Ida van der Ploeg, Linda Schild, Jan Koster, Evan E. Santo, Ellen M. Westerhout, Rogier Versteeg, Huib N. Caron, Jan J. Molenaar, M. Emmy M. Dolman

**Affiliations:** ^1^ Department of Oncogenomics, University of Amsterdam, Amsterdam, The Netherlands; ^2^ Department of Pediatric Oncology, Emma Kinderziekenhuis, Academic Medical Center, University of Amsterdam, Amsterdam, The Netherlands

**Keywords:** neuroblastoma, ABT199, BCL-2, MCL-1, resistance

## Abstract

The anti-apoptotic protein B cell lymphoma/leukaemia 2 (*BCL-2*) is highly expressed in neuroblastoma and plays an important role in oncogenesis. In this study, the selective BCL-2 inhibitor ABT199 was tested in a panel of neuroblastoma cell lines with diverse expression levels of BCL-2 and other BCL-2 family proteins. ABT199 caused apoptosis more potently in neuroblastoma cell lines expressing high BCL-2 and BIM/BCL-2 complex levels than low expressing cell lines. Effects on cell viability correlated with effects on BIM displacement from BCL-2 and cytochrome c release from the mitochondria. ABT199 treatment of mice with neuroblastoma tumors expressing high BCL-2 levels only resulted in growth inhibition, despite maximum BIM displacement from BCL-2 and the induction of a strong apoptotic response. We showed that neuroblastoma cells might survive ABT199 treatment due to its acute upregulation of the anti-apoptotic BCL-2 family protein myeloid cell leukaemia sequence 1 (MCL-1) and BIM sequestration by MCL-1. *In vitro* inhibition of MCL-1 sensitized neuroblastoma cell lines to ABT199, confirming the pivotal role of MCL-1 in ABT199 resistance. Our findings suggest that neuroblastoma patients with high BCL-2 and BIM/BCL-2 complex levels might benefit from combination treatment with ABT199 and compounds that inhibit MCL-1 expression.

## INTRODUCTION

Numerous cancer types have been associated with aberrations in genes encoding B cell lymphoma/leukaemia 2 (BCL-2) family proteins [1]. The BCL-2 family of proteins are key regulators of the intrinsic apoptotic pathway [2, 3], consisting of anti-apoptotic [e.g., BCL-2, BCL-extra large (BCL-X_L_), BCL-2-like protein 2 (BCL-W) and myeloid cell leukaemia sequence 1 (MCL-1)] and pro-apoptotic members [e.g., BCL-2-like protein 11 (BIM) and BH3 interacting domain death agonist (BID)] [4–6]. Increased interactions between anti-apoptotic and pro-apoptotic proteins inhibits apoptosis by preventing mitochondrial outer membrane permeabilization by the essential effector family members BAX and BAK. Consequently, cytochrome c cannot be released into the cytosol where it activates caspase 9-induced proteolysis and cell death [7–13].

Neuroblastoma is the most commonly diagnosed extracranial solid cancer in children, accounting for approximately 15% of all pediatric cancer deaths [14]. A large subset of neuroblastoma patients have enhanced levels of the anti-apoptotic gene *BCL-2* [15, 16]. Previously, we showed that selective *BCL-2* inhibition using RNA interference caused an apoptotic response in cell lines with moderate-to-high *BCL-2* levels. These findings could be confirmed with the small-molecule BCL-2 family inhibitor ABT263, which inhibits the anti-apoptotic activity of BCL-2, BCL-X_L_, BCL-W and MCL-1 with inhibition constant (K_i_) values of 0.044, 0.055, 7 and 224 nmol/L, respectively [17]. Neuroblastoma cell lines expressing high *BCL-2* levels responded better to ABT263 treatment than low *BCL-2*-expressing cell lines. ABT263 furthermore delayed the onset of tumor formation in mice injected with high *BCL-2*-expressing neuroblastoma cells [16]. These observations supported the potential benefit of BCL-2 family inhibitors for the future treatment of high *BCL-2*-expressing neuroblastoma tumors. Unfortunately, the administration of ABT263 in phase I/II clinical studies for adult cancers was associated with dose-limiting thrombocytopenia due to concomitant inhibition of anti-apoptotic BCL-X_L_, a key survival factor for circulating platelets [18–22]. Therefore, the more specific ABT263-derivative ABT199 was developed [17, 23, 24].

Compared with ABT263, ABT199 displays less activity against BCL-X_L_ (K_i_ of 48 nmol/L), BCL-W (K_i_ of 245 nmol/L) and MCL-1 (K_i_ of >444 nmol/L), while maintaining its activity against BCL-2 (K_i_ of <0.01 nmol/L) [17, 25]. ABT199 has shown preclinical and clinical efficacy against lymphoma, while sparing platelets [24, 26, 27]. In the current study, we explored the preclinical therapeutic potential of ABT199 for the treatment of BCL-2-dependent neuroblastoma tumors.

## RESULTS

### BCL-2 and BIM/BCL-2 complex levels predict sensitivity of neuroblastoma cells to ABT199

**Figure 1:**
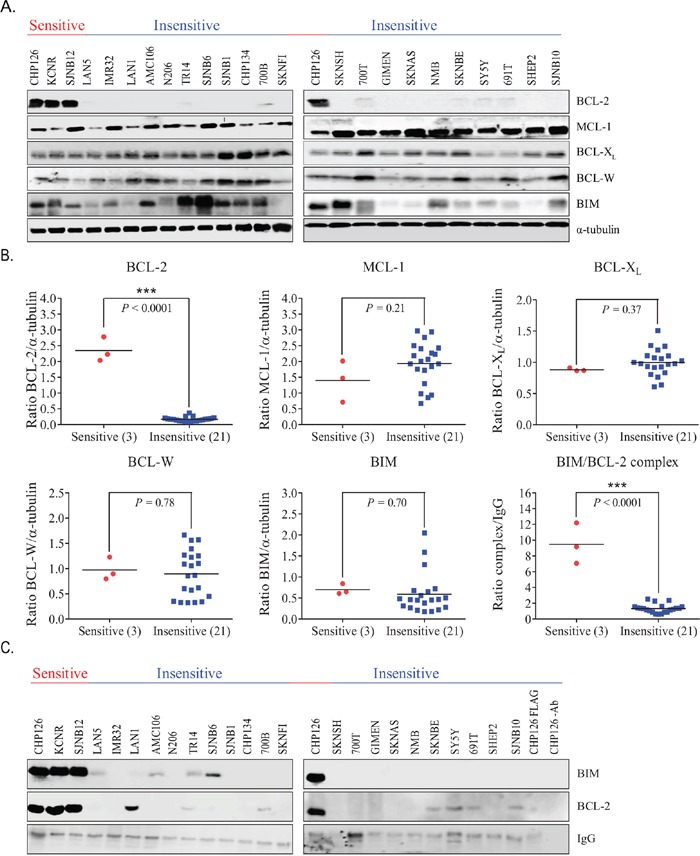
BCL-2 protein and BIM/BCL-2 complex levels predict sensitivity of neuroblastoma cell lines to ABT199 **A.** Western blot analysis of the protein expression levels of anti-apoptotic proteins BCL-2, MCL-1, BCL-X_L_, and BCL-W and the pro-apoptotic protein BIM in neuroblastoma cell lines. α-tubulin was used as household protein. **B.** protein levels of BCL-2, MCL-1, BCL-X_L_, BCL-W and BIM and BIM/BCL-2 complex levels in sensitive (i.e., CHP126, KCNR, and SJNB12) versus insensitive neuroblastoma cell lines. Protein levels shown in Figure A were quantified by calculating the protein/α-tubulin band intensity ratios. BIM/BCL-2 complex levels were established by anti-BCL-2 immunoprecipitation of whole cell lysates, followed by Western blotting for BIM. BIM band intensities were normalized to the IgG heavy chain of the BCL-2 antibody. Statistical differences between the sensitive and insensitive cell lines were calculated using a one-tailed (for BCL-2 and BIM/BCL-2 complex) or two-tailed unpaired Student *t* test, with *P* < 0.05 as the minimal level of significance and *P* < 0.0001 indicated as ^***^. Horizontal lines represent the mean of the relative intensities of the cell line panel. **C.** Western blot analysis of BIM/BCL-2 complex levels in 24 neuroblastoma cell lines, ordered from ABT199 sensitive (left) to ABT199 insensitive (right). The IgG heavy chain of the BCL-2 antibody served as a loading control. Total protein levels of the BCL-2-like family member proteins of the cell line panel in Figure 1A served as whole cell lysates for this experiment.

IC_50_ and LC_50_ values of ABT199 were established for 21 classical neuroblastoma cell lines and 3 tumor-initiating cell lines (TIC) (Table 1). Cell lines CHP126, KCNR and SJNB12 responded most potently to ABT199, with IC_50_ and LC_50_ values in the nanomolar range (i.e. 10-210 and 16-338 nmol/L, respectively) versus micromolar IC_50_ and LC_50_ values (i.e. 4.9-19.3 and 6.9-32.3 μmol/L, respectively) for the other cell lines tested. Evaluation of the BCL-2, MCL-1, BCL-X_L_, BCL-W and BIM protein levels (Figure 1A) showed significantly higher BCL-2 levels in the sensitive neuroblastoma cell lines CHP126, KCNR and SJNB12 compared with the insensitive cell lines (Figure 1B). No significant differences in expression of the anti-apoptotic proteins MCL-1, BCL-X_L_, BCL-W and the pro-apoptotic protein BIM were found between the sensitive and insensitive cell lines (Figure 1B). The same pattern was observed when looking at the mRNA level (Supplementary Figure S1A). Sensitive neuroblastoma cell lines expressed significantly higher *BCL-2* mRNA levels as compared to the insensitive cell lines, while no expression differences were observed for the other *BCL-2* family genes. Of note, BCL-2 protein levels better predict the sensitivity of neuroblastoma cell lines to ABT199 than *BCL-2* mRNA levels, as shown by the factor difference in average expression between the sensitive and insensitive cell lines (i.e. ∼14 versus ∼4). As ABT199 acts by displacing pro-apoptotic BCL-2 family members including BIM from BCL-2 [17], we also studied if levels of BIM bound to BCL-2 could be used as a predictive biomarker for sensitivity to ABT199. BCL-2 immunoprecipitation followed by immunoblotting for BIM showed that BIM/BCL-2 complex levels were indeed significantly higher in the sensitive neuroblastoma cell lines (Figure 1B and 1C). Results were confirmed by reciprocal co-immunoprecipitation experiments in which BIM/BCL-2 complex levels were determined by BIM immunoprecipitation followed by BCL-2 immunoblotting (Supplementary Figure S1A and S1B). We also tested if there was a correlation between ABT199 sensitivity and *MYCN* status. *MYCN*-amplified neuroblastoma cell lines responded more potently to ABT199 than *MYCN* single copy cell lines, with IC_50_ values ranging from 10 nmol/L-20 μmol/L versus 10-18 μmol/L, respectively (Supplementary Figure 1C).

**Table 1:** IC_50_ and LC_50_ values of ABT199 for neuroblastoma cell lines

Cell lines	IC_50_ (μmol/L)	LC_50_ (μmol/L)
CHP126	0.010	0.016
KCNR	0.026	0.153
SJNB12	0.210	0.338
LAN5	4.90	20.50
IMR32	6.01	8.80
AMC106	6.30	6.90
LAN1	6.40	7.10
N206	7.01	8.21
TR14	7.65	11.90
SJNB6	8.20	10.33
CHP134	9.40	17.70
SJNB1	9.40	9.50
700B	9.54	16.62
SKNFI	9.79	10.97
SKNSH	11.04	13.10
700T	11.29	17.50
GIMEN	14.01	21.80
SKNAS	14.20	19.10
NMB	14.60	17.40
SKNBE	14.70	18.60
SY5Y	15.32	17.71
691T	15.80	17.01
SHEP2	18.30	21.60
SJNB10	19.30	32.30

### ABT199 causes cell death in BCL-2-dependent neuroblastoma cells by activation of the intrinsic apoptotic program

**Figure 2:**
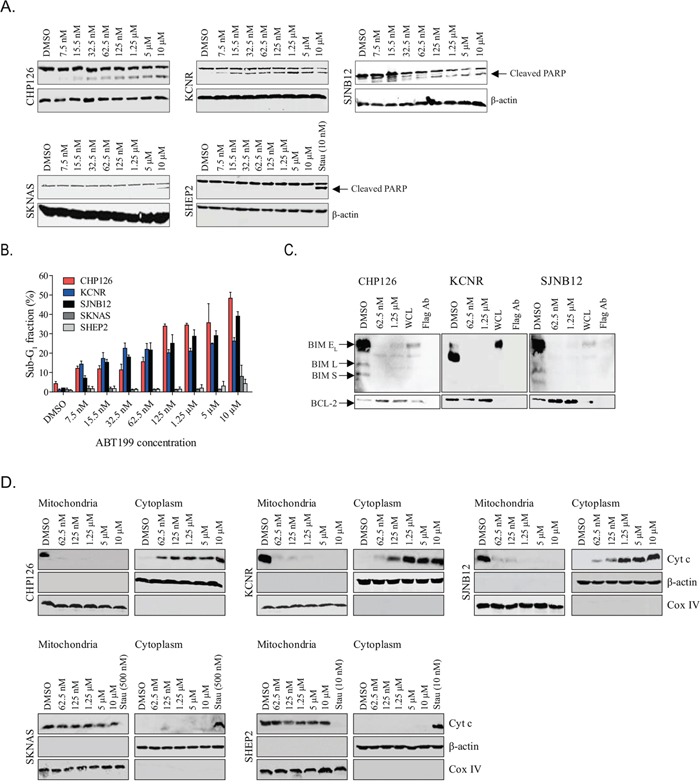
ABT199 induces cell death in BCL-2 dependent neuroblastoma cell lines through activation of the intrinsic apoptotic pathway **A.** Western blot analysis of the *in vitro* effects of ABT199 on PARP cleavage after 72-hour treatment of sensitive neuroblastoma cell lines CHP126, KCNR and SJNB12 and insensitive cell lines SKNAS and SHEP2 with increasing ABT199 concentrations. β-actin served as loading control. As ABT199 failed to induce PARP cleavage in SHEP2, staurosporine (10 nmol/L, 24 hours; indicated as Stau) was used as a positive control for this cell line. **B.** FACS analysis of the *in vitro* effects on sub-G_1_ induction after 72-hour treatment with increasing ABT199 concentrations. Data represent the mean percentages of cells in sub-G_1_ ± SD of three replicate experiments. **C.** *in vitro* effects on BIM displacement from BCL-2 after 24-hour treatment of CHP126, KCNR and SJNB12 with 62.5 nmol/L or 1.25 μmol/L ABT199. BIM displacement was established by detecting BIM/BCL-2 complex levels by anti-BCL-2 immunoprecipitation, followed by Western blotting for BIM. BCL-2 levels served as loading control. (BIM E_L_ = BIM extra-large, BIM L = BIM large and BIM S = BIM small) WCL= whole cell lysate. **D.** Western blot analysis of the *in vitro* effects of ABT199 on cytochrome c release from the mitochondria into the cytosol. Cytochrome c levels (indicated as Cyt c) in the mitochondrial and cytoplasmic cell fractions were established after 24-hour treatment of the cell lines with increasing ABT199 concentrations. COX IV and β-actin were used as loading controls for the mitochondrial and cytoplasmic fractions, respectively. As ABT199 failed to induce cytochrome c release in SKNAS and SHEP2, cell lines were 24-hour treated with 500 and 10 nmol/L staurosporine, respectively, as a positive control.

High BCL-2-expressing neuroblastoma cell lines CHP126, KCNR and SJNB12 and low BCL-2-expressing cell lines SKNAS and SHEP2 were treated with increasing doses of ABT199 to study effects on apoptosis. PARP cleavage induction in the high BCL-2-expressing cell lines was already observed after treatment with only 7.5 nmol/L ABT199, while for the low BCL-2-expressing neuroblastoma cell lines PARP cleavage was only detected in SKNAS after treatment with 10 μmol/L ABT199 (Figure 2A). Similar results were obtained when investigating the effects of ABT199 on cleaved caspase 3 (Supplementary Figure S2A). Apoptotic effects of ABT199 were further validated by flow cytometry. In line with the effects on PARP and caspase 3 cleavage, ABT199 treatment of the high BCL-2-expressing neuroblastoma cell lines resulted in more pronounced increases in sub-G_1_ fraction than observed for the low BCL-2-expressing cell lines (Figure 2B and Supplementary Table S1) Treatment with only 7.5 nmol/L ABT199 resulted in increases in sub-G_1_ fraction of 8% (CHP126), 13% (KCNR) and 5% (SJNB12) for the high BCL-2-expressing cell lines versus 0% (SKNAS) and 1% (SHEP2) for the low BCL-2-expressing cell lines. Effects on sub-G_1_ were dose-dependent, with maximum increases observed after treatment with 10 μmol/L ABT199 (i.e. 44%, 25% and 37% for CHP126, KCNR and SJNB12, respectively, versus 7% and 3% for SKNAS and SHEP2, respectively). Next, *in vitro* effects of ABT199 on the activation of the intrinsic apoptotic pathway were studied. As ABT199 inhibits the activity of BCL-2 by displacement of pro-apoptotic proteins, we first studied the effects of ABT199 on BIM displacement from BCL-2. Treatment of the high BCL-2-expressing neuroblastoma cell lines with 62.5 nmol/L ABT199 was already sufficient for almost complete displacement of BIM from BCL-2 (Figure 2C). No or only moderate increases in BIM displacement were observed after treatment with higher ABT199 concentrations (i.e. 1.25 μmol/L). BCL-2-dependent activation of the intrinsic apoptotic program was further studied by evaluation of the effects of ABT199 on cytochrome c release. Dose-dependent cytochrome c release from the mitochondria into the cytoplasm was observed after ABT199 treatment of CHP126, KCNR and SJNB12 (Figure 2D). In line with the effects on BIM displacement from BCL-2, cytochrome c release was already observed after treatment with nanomolar concentrations of ABT199. No cytochrome c release was observed after ABT199 treatment of the low BCL-2-expressing cell lines, even at the highest concentration of 10 μmol/L (Figure 2D). Together, these findings confirm that ABT199 causes apoptosis in BCL-2-dependent neuroblastoma cells via activation of the intrinsic apoptotic pathway. This was strengthened by the observation that the effects of ABT199 on PARP and caspase 3 cleavage and sub-G_1_ fraction could be completely rescued in KCNR by combination treatment with the pan-caspase inhibitor QVD-OPH (Supplementary Figure S2D and S2E).

In addition, overexpression of BCL-2 in the low BCL-2-expressing neuroblastoma cell line SY5Y resulted in a strong increase in sensitivity to SY5Y (Supplementary Figure S2B and S2C). The remarkable decrease in IC_50_ value observed after BCL-2 overexpression (i.e. from 16.3 μM to 2.6 μM) shows that the effects of ABT199 obtained in CHP126, KCNR and SJNB12 are indeed caused by BLC-2 inhibition rather than off-target effects.

### ABT199 causes apoptosis in high BCL-2-expressing neuroblastoma xenografts by BIM displacement from BCL-2

**Figure 3:**
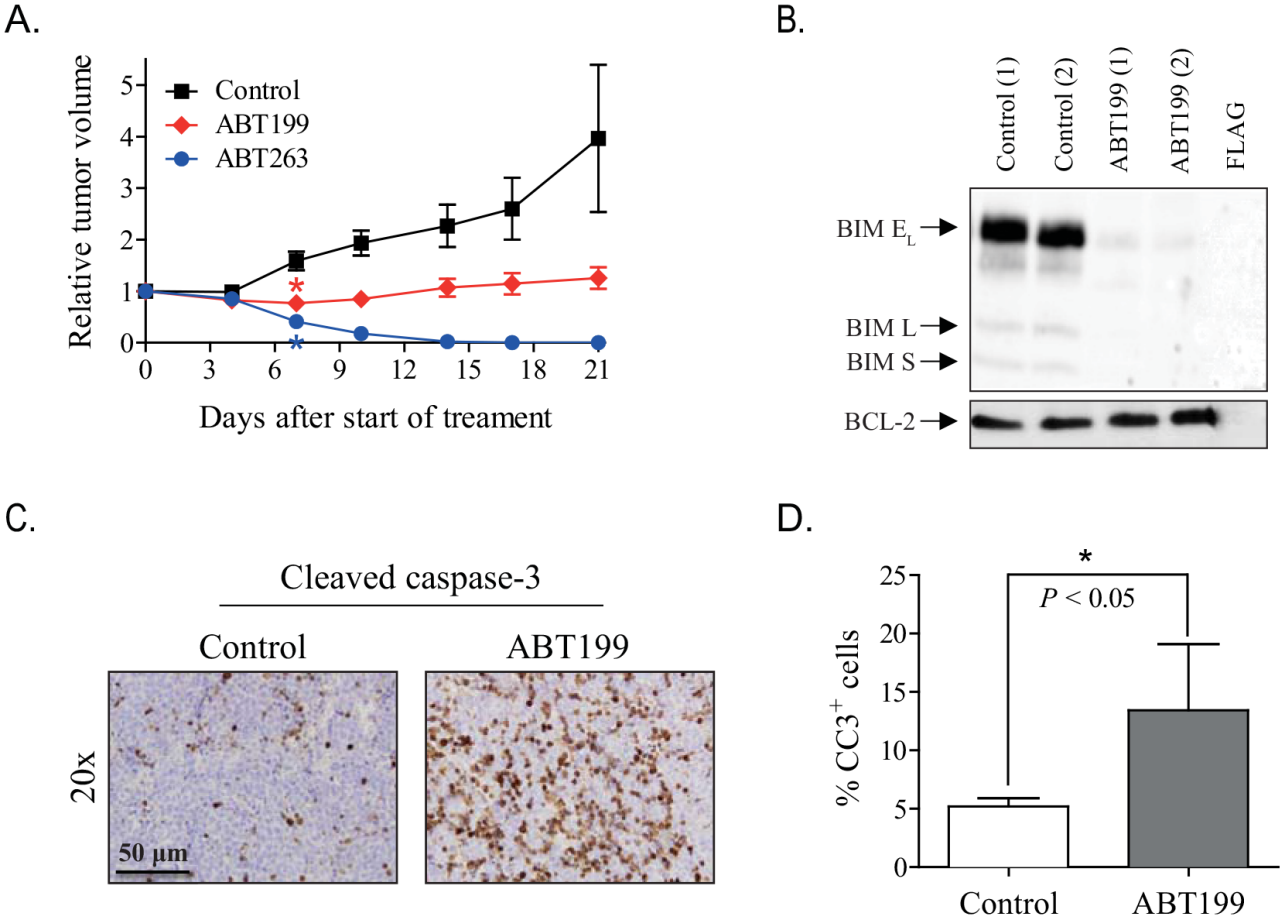
ABT199 and ABT263 cause apoptosis in mice with KCNR neuroblastoma xenografts expressing high levels of BCL-2 and BIM/BCL-2 complex **A.** inhibitory effects of ABT199 and ABT263 on the growth of KCNR neuroblastoma xenografts in mice. Relative tumor volume was calculated as the volume at the indicated day after start of treatment divided by the volume prior to treatment initiation. Data represent the mean relative tumor volume ± SEM [Group sizes: *n* = 10 (control; 10% ethanol/30% PEG 400/60% phosal 50 PG (v/v/v)), *n* = 5 (100 mg/kg ABT199), and *n* = 5 (100 mg/kg ABT263)]. Statistical differences between treated and control groups were calculated using one-way ANOVA Bonferroni adjustment and are indicated on the first day after treatment initiation at which a statistically different effect was observed (*). *P* values < 0.05 were considered significant. **B.** *in vivo* effects of ABT199 on BIM displacement from BCL-2. Effects were studied by detecting BIM/BCL-2 complex levels by anti-BCL-2 immunoprecipitation, followed by Western blotting for BIM (BIM E_L_ = BIM extra-large, BIM L = BIM large and BIM S = BIM small). Levels of BIM/BCL-2 complex were established for *n* = 2 mice per group at 4 hours after administration of the last dose of ABT199. BCL-2 levels served as loading control. Total protein levels of the BCL-2 family members in Figure 4D served as input levels for this experiment. **C.** representative microscopic images of cleaved caspase 3 stained paraffin-embedded tumor sections of control and ABT199-treated tumor samples collected at 4 hours after administration of the last dose. Magnification of the images: 20x. Scale bar: 50 μm. **D.** quantification of the stimulatory effect of ABT199 on cleaved caspase 3 was performed by manual counting of the total number of cells and the number of cleaved caspase 3-positive (CC3^+^) cells in 20 microscopic fields per mouse sample at 40x magnification (30% of each field). The average percentage of CC3^+^ cells for each mouse was used to determine the effect of ABT199. Statistical differences between the control group (*n* = 6) and ABT199-treated group (*n* = 2) were calculated using a one-tailed unpaired Students *t* test and are indicated as * (*P* < 0.05).

The efficacy of ABT199 was subsequently studied *in vivo* in mice with high BCL-2-expressing KCNR neuroblastoma xenografts. Once-daily oral treatment with 100 mg/kg ABT199 for three consecutive weeks resulted in significant tumor growth inhibition relative to the vehicle treated controls (Figure 3A). However, comparison studies in the same animal model demonstrated superior antitumor activity of ABT263 over ABT199, i.e., complete tumor regression versus tumor growth inhibition (Figure 3A).

The difference in efficacy between ABT199 and ABT263 might be the result of the concomitant inhibition of other anti-apoptotic BCL-2 family members by ABT263 and/or incomplete BCL-2 inhibition by ABT199. We therefore studied the *in vivo* effects of ABT199 on BIM displacement from BCL-2. Co-immunoprecipitation studies at 4 h after administration of the last ABT199 dose showed almost complete release of BIM, while in the control mice BIM was still firmly complexed to BCL-2 (Figure 3B). This indicates that sufficient high intratumoral ABT199 levels were achieved for maximum target inhibition. In line with ABT199 effects on BIM displacement from BCL-2, a strong apoptotic response was observed. Treatment with 100 mg/kg/day ABT199 caused an increase in cleaved caspase 3-positive cells (Figure 3C and 3D). No clear phenotypic changes and effects on cell proliferation were observed, as shown by hematoxylin-eosin and Ki67 staining of tumor tissues, respectively (Supplementary Figure S3A). Together, these results show that the differential efficacy between ABT199 and ABT263 is not the result of insufficient target inhibition by ABT199, but might result from simultaneous inhibition of multiple anti-apoptotic BCL-2 family members by ABT263. Discontinuation of treatment with ABT199 and ABT263 resulted in tumor growth and tumor recurrence, respectively (Supplementary Figure S3B).

### MCL-1 stabilization and upregulation and BIM sequestration by MCL-1 provide a rationale for combination treatment with MCL-1 inhibitors

**Figure 4:**
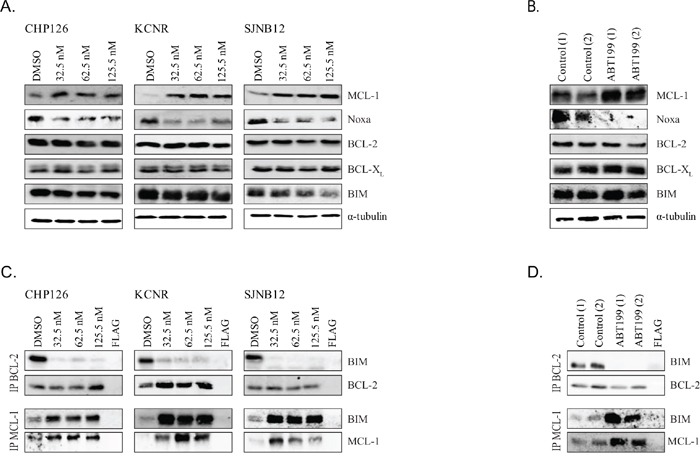
ABT199 causes MCL-1 upregulation and Noxa downregulation, leading to increased sequestration of released BIM from BCL-2 **A.** *in vitro* effects of ABT199 on total protein levels of MCL-1, Noxa, BCL-2, BCL-X_L_ and BIM in CHP126, KCNR and SJNB12 after 24-hour treatment with increasing doses. Effects were determined by Western blot analysis using α-tubulin as a loading control. **B.** *in vivo* effects of ABT199 on total protein levels of MCL-1, Noxa, BCL-2, BCL-X_L_ and BIM. Mice with KCNR neuroblastoma xenografts were daily treated with vehicle or 100 mg/kg ABT199 for 3 consecutive weeks. Tumor samples were collected at 4 hours after administration of the last dose and analyzed by Western blot analysis. α-tubulin served as a loading control. **C.** *in vitro* effects of ABT199 on BIM/BCL-2, and BIM/MCL-1 complex levels in CHP126, SJNB12 and KCNR after 24-hour treatment with increasing doses. BIM/BCL-2 and BIM/MCL-1 complex levels were established by immunoprecipitation of BCL-2 and MCL-1 followed by Western blot analysis of BIM. Immunoprecipitated levels of BCL-2 and MCL-1 were used as loading controls. **D.** *in vivo* effects of ABT199 on BIM/BCL-2 and BIM/MCL-1 complex levels. Tumor samples (*n* = 2 per group) were collected after 3 weeks treatment with vehicle or 100 mg/kg/day ABT199, at 4 hours after administration of the last dose. BIM/BCL-2 and BIM/MCL-1 complex levels were subsequently determined as described above.

Upregulation and increased activity of the non-targeted anti-apoptotic BCL-2 family proteins MCL-1 and BCL-X_L_ have been reported as key mechanisms of resistance to BCL-2 inhibitors including ABT199 [28–33]. We observed that ABT199 treatment of the high BCL-2-expressing neuroblastoma cell lines CHP126, KCNR and SJNB12 indeed resulted in strongly increased MCL-1 levels, while the other anti-apoptotic proteins and the pro-apoptotic protein BIM were not affected (Figure 4A). As Noxa is a negative regulator of MCL-1 [33], effects of ABT199 on Noxa protein levels were also studied. Noxa protein levels were decreased after ABT199 treatment of CHP126, KCNR and SJNB12 (Figure 4A), which might explain the increased MCL-1 levels. Real-time quantitative PCR analysis after 24 h treatment of KCNR cells treated with increasing ABT199 concentrations showed no significant changes in *MCL-1* and *Noxa* mRNA levels (Supplementary Figure S3C and S3D). *In vitro* observations were confirmed in the *in vivo* experiments using tumor materials of the ABT199 treated KCNR xenografts and control samples. MCL-1 protein levels were again strongly increased after ABT199 treatment, while Noxa was decreased (Figure 4B). No effects of ABT199 on anti-apoptotic proteins BCL-2 and BCL-X_L_ and the pro-apoptotic protein BIM were observed *in vivo*. Affymetrix mRNA profiling of the *in vivo* tumor samples revealed no significant changes in *BCL-2*, *MCL-1*, *BCL-X_L_* and *BIM* mRNA levels after ABT199 treatment (Supplementary Figure S3E). The lack of effects on *MCL-1* mRNA indicates that ABT199-induced MCL-1 upregulation is caused at the protein rather than mRNA level, making the increased stabilization of MCL-1 by reduced Noxa protein levels more plausible. While also not statistically significant, *in vivo Noxa* mRNA levels appeared to be lower after ABT199 treatment. Next, the *in vitro* sequestration of released BIM by other anti-apoptotic proteins was studied. After 24 h treatment of neuroblastoma cell lines CHP126, KCNR and SJNB12 with nanomolar concentrations ABT199, shifting of BIM from BCL-2 to the anti-apoptotic protein MCL-1 was observed while no sequestration of released BIM by the anti-apoptotic protein BCL-X_L_ was observed. (Figure 4C and Supplementary Figure S3F). BIM displacement from BCL-2 to MCL-1 also occurred *in vivo* (Figure 4D), confirming the pivotal role of MCL-1 in the biological mechanism by which neuroblastoma cells can escape from ABT199-induced apoptosis.

**Figure 5:**
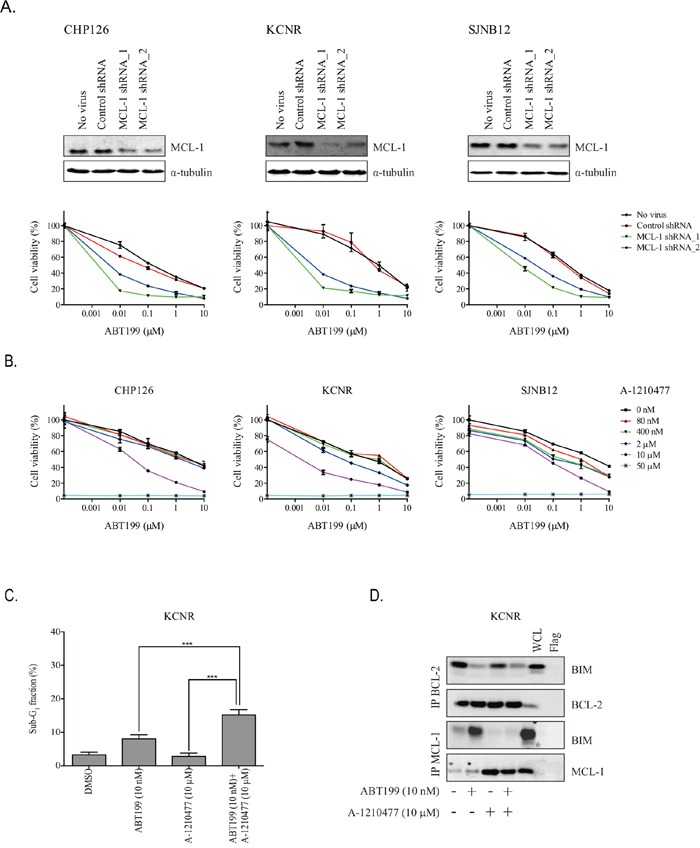
MCL-1 inhibition sensitizes high BCL-2 and BIM/BCL-2-expressing neuroblastoma cells to ABT199 **A.** *in vitro* effects of MCL-1 knockdown on the sensitivity of neuroblastoma cell lines CHP126, KCNR and SJNB12 to ABT199. Cells were 72-hour transfected with non-targeting control shRNA or MCL-1 shRNA (i.e., MCL-1 shRNA_1 or MCL-1 shRNA_2) prior to treatment with DMSO (control) or indicated ABT199 concentrations. Cell viability was assessed after 72-hour treatment using the MTT colorimetric assay. For each cell line, the viability of non-transfected cells treated with DMSO was set to 100%. **B.** *in vitro* effects of the small molecule MCL-1 inhibitor A-1210477 on the sensitivity of CHP126, KCNR and SJNB12 to ABT199. Cells were co-treated with 0-10 μmol/L ABT199 and 0-50 μmol/L A-1210477. Effects on cell viability were established after 72-hour treatment using the MTT colorimetric assay. For each cell line, the viability of untreated cells was set to 100%. **C.** FACs analysis of KCNR after 72-hour treatment with 10 nmol/L ABT199 alone or in combination with 10 μmol/L A-1210477. Data represent the mean percentages of cells in sub-G_1_ ± SD of three replicate experiments. **D.** *in vitro* effects on BIM displacement from BCL-2 and MCL-1 after 24-hour treatment of KCNR with 10 nmol/L of ABT199 or 10μmol/L of A-1210477 and a combination of both compounds. BIM displacement was established by detecting BIM/BCL-2 and BIM/MCL-1 complex levels by anti-BCL-2 and anti-MCL-1 immunoprecipitation, followed by Western blotting for BIM. BCL-2 and MCL-1 levels served as loading control.

To further demonstrate the importance of MCL-1 in neuroblastoma resistance to ABT199, combined effects of *MCL-1* knockdown and ABT199 on cell viability were studied. *MCL-1* knockdown in CHP126, KCNR and SJNB12 using two different shRNAs sensitized the neuroblastoma cell lines to ABT199 treatment (Figure 5A). Effects of MCL-1 inhibition on the sensitivity of neuroblastoma cell lines CHP126, KCNR and SJNB12 to ABT199 were additionally studied using the selective MCL-1 inhibitor A-1210477. Combined treatment of CHP126, KCNR and SJNB12 with ABT199 and A-1210477 caused additive to slightly synergistic inhibitory effects on cell viability (Figure 5B and Table 2). In line with the effects on cell viability, combination treatment of KCNR with ABT199 and A-1210477 resulted in a significantly larger increase in sub-G_1_ fraction than treatment with either of the inhibitors alone (Figure 5C). BCL-2/BIM co-immunoprecipitation studies confirmed the occurrence of BIM displacement from BCL-2 after treatment with ABT199 alone or in combination with A-1210477. In a similar manner, MCL-1/BIM co-immunoprecipitation studies showed that the increased binding of BIM/to MCL-1 observed after monotherapy with ABT199 was prevented by combination treatment with A-1210477 (Figure 5D). Taken together, these results demonstrate that the efficacy of ABT199 can be potentiated by preventing sequestration of released BIM by MCL-1.

**Table 2:** Synergy between BCL-2 inhibitor ABT199 and MCL-1 inhibitor A-1210477 in high BCL-2-expressing neuroblastoma cell lines

	CHP126	KCNR	SJNB12
A-1210477	++++ (0.18)	+++ (0.28)	+ (0.74)

Combination indices (between brackets) were determined according to Chou and Talalay [44].

## DISCUSSION

Based on previously performed preclinical studies [16], the BCL-2 inhibitor ABT263 was considered to have high potential for testing in neuroblastoma patients. Unfortunately, the clinical implementation of ABT263 was hampered because of dose-limiting thrombocytopenia due to concomitant inhibition of the anti-apoptotic protein BCL-X_L_. The current manuscript describes the preclinical evaluation of the novel selective BCL-2 inhibitor ABT199. In line with previous results obtained with ABT263, neuroblastoma cell lines expressing high *BCL-2* mRNA levels responded more potently to ABT199 than low *BCL-2*-expressing cell lines, with an over 90-fold difference in average LC_50_ value (i.e., 0.17 μM versus 15.46 μM, respectively). *In vitro* efficacy studies furthermore showed that BCL-2 protein and the BIM/BCL-2 complex levels are better predictive biomarkers for ABT199 sensitivity than *BCL-2* mRNA levels. Although less strong, MYCN amplification status was also observed to be a predictive biomarker for sensitivity to ABT199.

The current manuscript describes the first side-by-side *in vivo* comparison of ABT199 with ABT263 for solid cancer treatment. Although ABT199 induced a strong apoptotic response, comparison of the efficacy of equal doses of ABT199 and ABT263 in mice with KCNR neuroblastoma xenografts expressing high levels of BCL-2 showed superior antitumor activity for ABT263. As maximum BIM displacement from BCL-2 was observed after ABT199 treatment, the most plausible explanation for the superior efficacy of ABT263 is its additional activity against anti-apoptotic proteins BCL-X_L_ and BCL-W. Results indicate that the simultaneous inhibition of multiple anti-apoptotic proteins is more effective for treatment of BCL-2-dependent neuroblastoma than inhibition of BCL-2 alone.

The importance of inhibiting multiple anti-apoptotic proteins is strengthened by the observations that *in vitro* and *in vivo* treatment with ABT199 resulted in upregulation of the anti-apoptotic protein MCL-1 and sequestration of released BIM by MCL-1. Upregulation of non-targeted anti-apoptotic BCL-2 family proteins taking over the function of BCL-2 has earlier been described as one of the key mechanisms of tumor resistance to BCL-2 inhibitors including ABT199 [28–32, 34]. Long-term ABT199 treatment of non-Hodgkin lymphoma cells resulted in upregulated levels of anti-apoptotic proteins MCL-1 and BCL-X_L_ that sequestered BIM [29]. No changes in the expression and activity of BCL-X_L_ were observed after ABT199 treatment, indicating that MCL-1 plays a more important role in neuroblastoma resistance to ABT199 than BCL-X_L_.

MCL-1 upregulation after ABT199 treatment could be explained by Noxa downregulation and MCL-1 stabilization by BIM. Binding of Noxa to MCL-1 triggers Mule-mediated ubiquitination and proteasomal degradation of the anti-apoptotic protein, resulting in decreased MCL-1 levels [33, 35–40]. We observed that Noxa was downregulated after *in vitro* and *in vivo* treatment with ABT199, which has not been reported for BCL-2 inhibitors before. Since complexation of BIM with MCL-1 stabilizes MCL-1 independent of Noxa [33], BIM displacement from BCL-2 to MCL-1 after ABT199 treatment might also contribute to upregulated MCL-1 levels.

Combining *MCL-1* knockdown with ABT199 treatment resulted in synergistic cell growth inhibition. Potentiating effects of MCL-1 inhibition were confirmed with the small molecule MCL-1 inhibitor A-1210477, but combined effects of ABT199 and A-1210477 were additive rather than synergistic. The less potentiating effects obtained with A-1210477 might be due to the incomplete displacement of BIM from MCL-1, which has been described before. In addition, MCL-1 might have other functions independent of its role in apoptosis such as maintenance of mitochondrial integrity and deregulation of mitochondrial integrity by *MCL-1* knockdown in combination with BCL-2 inhibitors might have a more lethal effect on neuroblastoma cells than inhibition of the pro-apoptotic function of MCL-1 alone. Of interest is the observation that several compounds in use or development for cancer treatment also cause MCL-1 inhibition (e.g., CDK inhibitors, MEK inhibitors and PI3K/mTOR inhibitors [24, 28, 29]. Specific combination studies with these types of compounds should be considered.

For the clinical use of ABT199 it is important to identify pharmacodynamic biomarkers for efficacy. In line with previously published results for ABT199 [17], our study demonstrated that BIM displacement from BCL-2 and cytochrome c release from the mitochondria can be utilized as target-specific efficacy biomarkers for ABT199. *In vitro* dose-dependent stimulatory effects on PARP- and caspase 3 cleavage and the *in vivo* observed cleaved caspase 3 induction after ABT199 treatment furthermore indicate that both apoptotic markers are potential non-target specific biomarkers for ABT199 efficacy. Additional studies are required to validate the dose-dependency of all potential biomarkers *in vivo*.

Taken together, the results presented in this study strongly suggest that children with neuroblastoma tumors expressing high levels of BCL-2 and the BIM/BCL-2 complex might benefit from combined treatment with ABT199 and compounds that inhibit MCL-1.

## MATERIALS AND METHODS

### Chemicals

ABT199, ABT263 and A-1210477 were purchased from Selleck Chemicals while QVD-OPH was purchased from Sigmaaldrich. For *in vivo* studies ABT199 and ABT263 were formulated in 10% ethanol/30% polyethylene glycol (PEG) 400/60% phosal 50 propylene glycol (PG) (v/v/v) in final concentrations of 10 mg/mL.

### Cell culture

Classical human neuroblastoma cell lines and neuroblastoma tumor-initiating cell (TIC) lines were cultured as previously described [41, 42]. Cell culture protocols are described in detail in the Supplementary Materials and Methods.

### IC_50_ and LC_50_

Neuroblastoma cell lines were seeded in triplicate in 96-well (classical cell lines) or 48-well (TIC lines) plates using the most optimal confluency for each cell line [42]. Cells were incubated overnight and treated with 1 nmol/L to 50 μmol/L ABT199. Control samples were treated with 0.5% DMSO. Cell viability was determined prior to and after 72-hour treatment using the 3-(4.5-dimethylthiazol-2-yl)-2,5-diphenyltetrazolium bromide (MTT) colorimetric assay [43]. Half maximal effective concentration (IC_50_) and half lethal concentration (LC_50_) values were derived from dose-response curves. IC_50_ values at 72 hours were calculated by determining the ABT199 concentrations needed to achieve a 50% reduction in cell viability observed for DMSO-treated cells at 72 hours (set at 100%). LC_50_ values at 72 hours were calculated by establishing the ABT199 concentrations needed to attain a 50% reduction in the cell viability compared to time point 0.

### FACS analysis

Cells were treated with 0.1% DMSO (control) or ABT199 using concentration ranges of 7.8 nmol/L to 10 μmol/L. After 72-hour treatment, floating and adherent cells were harvested for FACS analysis to determine the cell-cycle distribution and the apoptotic sub-G_1_ fraction. See Supplementary Materials and Methods for a detailed protocol.

### Cell fractionation

CHP126, KCNR, SJNB12, SKNAS and SHEP2 were 24-hour treated with 0.04% DMSO (control) or 62.5 nmol/L to 10 μmol/L ABT199. Floating and adherent cells were harvested to determine cytochrome c in the cytosolic and organelle fractions. See Supplementary Materials and Methods for a detailed protocol.

### *In vitro* western blotting

The following antibodies were used: rabbit anti-human BCL-2 (clone D55G8) monoclonal antibody (1:1,000, Cell Signaling Technology); rabbit anti-human BCL-X_L_ (clone 54H6) monoclonal antibody (1:1,000, Cell Signaling Technology); rabbit anti-human MCL-1 monoclonal antibody (1:1,000, Cell Signaling Technology); rabbit anti-human BCL-W (Clone 31H4) monoclonal antibody (1:1,000, Cell Signaling Technology); rabbit anti-human Noxa (clone EPR9735B) monoclonal antibody (1:1,000, Abcam); rabbit anti-human BIM (1:1,000, Cell Signaling Technology); mouse anti-human cytochrome c (clone 7H8.2C12) monoclonal antibody (1:1,000 BD pharmingen); rabbit anti-human PARP (Clone 9542S) monoclonal antibody (1:1,000, Cell Signaling Technology); rabbit anti-human cleaved caspase 3 (Clone 5AE17) monoclonal antibody (1:1000, Cell Signaling Technology); mouse anti-human α-tubulin (clone DM1A) monoclonal antibody (1:10,000, Cell Signaling Technology) and horseradish peroxidase (HRP)-conjugated goat anti-rabbit (clone NA9340V) and goat anti-mouse (clone NXA931) secondary antibodies (1:10,000 GE Healthcare). See Supplementary Materials and Methods for detailed protocol.

### *In vitro* co-immunoprecipitation and immunoblotting

Cell lines were seeded onto 14-cm culture dishes. For the detection of basal BIM/BCL-2 complex levels, untreated cells were harvested after 72 hours incubation at normal culture conditions. For the detection of BIM displacement from BCL-2 and BIM complexation with MCL-1 and BCL-X_L_, cells were treated with 0.25% DMSO (control) or 62.5 nmol/L or 1.25 μmol/L ABT199 at 24 hours after seeding and harvested after 24-hour treatment. Co-immunoprecipitation studies have been performed as described in detail in the Supplementary Materials and Methods.

### Cell transfection

CHP126, KCNR, SJNB12 and SY5Y were seeded in 6-cm culture dishes (2×10^5^ cells in 4 mL culture medium) and incubated overnight. Next, cells were transfected with non-targeting shRNA (AACAAGATGAAGAGCACCAA; negative control) or MCL-1 shRNA (TRCN0000199070 and TRCN0000005518) for the BCL-2 high-expressing cell lines using the PLenti VI system according to the manufacturers protocol (Sigma Aldrich), while SY5Y was transfected with either a luciferase 2 control or a pLenti 6/V5-DEST vector constitutively overexpressing the BCL-2 protein. After 72 hours, cells were transferred into 96-well plates for *in vitro* MTT assays and synergy studies (see below).

### *In vitro* synergy assays

CHP126, KCNR and SJNB12 transiently transfected with non-targeting shRNA or *MCL-1* shRNA were seeded in duplicates in 96-well plates and incubated overnight. Cells were then treated with ten-fold serial dilutions of ABT199 (0-10 μmol/L). Effects on cell viability were studied after 72-hour treatment with ABT199, using the MTT cell proliferation assay.

For synergy studies between ABT199 and the MCL-1 inhibitor A-1210477, non-transfected cells in 96-well plates were co-treated with ten-fold serial dilutions of ABT199 (0-10 μmol/L) and five-fold serial dilution of A-1210477 (0-50 μmol/L).

### *In vivo* efficacy in neuroblastoma mouse models

Female NMRI *nu*^−^/*nu*^−^ mice (6-15 weeks old; 20-30 g) were obtained from Harlan) and experiments were performed with permission from and according to the standards of the Dutch animal ethics committee (DAG 102776, 102830 and 102690). NMRI *nu*^−^/*nu*^−^ mice with KCNR neuroblastoma xenografts of approximately 268 mm^3^ were orally treated with 100 mg/kg/d ABT199 (n = 5), 100 mg/kg/d ABT263 (n = 5), or vehicle (n = 6) for 21 days. Tumor sizes were measured by an external caliper. See Supplementary Materials and Methods for a more detailed protocol.

### *In vivo* western blotting

Per mouse sample, 10 tumor sections of 50 μm were homogenized in 2% Chaps buffer as previously described. Western blot detection of protein levels of BCL-2 like family members was carried out as described for *in vitro* Western blotting.

### *In vivo* co-immunoprecipitation

Sections of treated and untreated KCNR tumors harvested at 4 hours after administration of the last dose were homogenized using the Ultra Turrax T25 tissue homogenizer (Janke & Kunkel) and lysed (overnight at 4°C) in 2% Chaps buffer. Co-immunoprecipitation was carried out as described above.

### *In vivo* immunohistochemistry

The following antibodies were used: rabbit anti-human Ki-67 (clone SP6) monoclonal antibody (1:1,000, Thermo Scientific), rabbit anti-human cleaved caspase 3 (Asp175) polyclonal antibody (1:100, Cell Signaling Technology) and BrightVision horseradish peroxidase-conjugated goat anti-rabbit polyclonal secondary antibody (undiluted; 30 min; Immunologic). See Supplementary Materials and Methods for a detailed protocol.

### mRNA expression profiling

RNA was extracted from tumors with TRIzol (Invitrogen, Carlsbad, CA) following the manufacturers protocols. RNA concentration and quality were determined using the RNA 6000 Nano assay on the Agilent 2100 Bioanalyzer (Agilent Technologies). Fragmentation of cRNA, hybridization to hg-u133 plus 2.0, microarrays and scanning were carried out according to the manufacturers protocol (Affymetrix inc. Santa Barbara, CA). The mRNA gene expression data were normalized with the MAS5.0 algorithm within the GCOS program of Affymetrix Inc. Target intensity was set to 100. All data were analyzed using the R2 genomic analysis and visualization platform (http://r2.amc.nl).

## SUPPLEMENTARY MATERIALS FIGURES AND TABLE



## References

[1] Yip KW, Reed JC (2008). Bcl-2 family proteins and cancer. Oncogene.

[2] Czabotar PE, Lessene G, Strasser A, Adams JM (2014). Control of apoptosis by the BCL-2 protein family: implications for physiology and therapy. Nat Rev Mol Cell Biol.

[3] Iqbal J, Neppalli VT, Wright G, Dave BJ, Horsman DE, Rosenwald A, Lynch J, Hans CP, Weisenburger DD, Greiner TC, Gascoyne RD, Campo E, Ott G, Muller-Hermelink HK, Delabie J, Jaffe ES (2006). BCL2 expression is a prognostic marker for the activated B-cell-like type of diffuse large B-cell lymphoma. J Clin Oncol.

[4] Chipuk JE, Moldoveanu T, Llambi F, Parsons MJ, Green DR (2010). The BCL-2 family reunion. Mol Cell.

[5] Green DR, Kroemer G (2004). The pathophysiology of mitochondrial cell death. Science.

[6] Llambi F, Moldoveanu T, Tait SW, Bouchier-Hayes L, Temirov J, McCormick LL, Dillon CP, Green DR (2011). A unified model of mammalian BCL-2 protein family interactions at the mitochondria. Mol Cell.

[7] Davids MS, Letai A (2012). Targeting the B-cell lymphoma/leukemia 2 family in cancer. J Clin Oncol.

[8] Hu W, Kavanagh JJ (2003). Anticancer therapy targeting the apoptotic pathway. Lancet Oncol.

[9] Korsmeyer SJ, Wei MC, Saito M, Weiler S, Oh KJ, Schlesinger PH (2000). Pro-apoptotic cascade activates BID, which oligomerizes BAK or BAX into pores that result in the release of cytochrome c. Cell Death Differ.

[10] Saelens X, Festjens N, Vande Walle L, van Gurp M, van Loo G, Vandenabeele P (2004). Toxic proteins released from mitochondria in cell death. Oncogene.

[11] Sun XM, MacFarlane M, Zhuang J, Wolf BB, Green DR, Cohen GM (1999). Distinct caspase cascades are initiated in receptor-mediated and chemical-induced apoptosis. J Biol Chem.

[12] Tait SW, Green DR (2010). Mitochondria and cell death: outer membrane permeabilization and beyond. Nat Rev Mol Cell Biol.

[13] Wei MC, Lindsten T, Mootha VK, Weiler S, Gross A, Ashiya M, Thompson CB, Korsmeyer SJ (2000). tBID, a membrane-targeted death ligand, oligomerizes BAK to release cytochrome c. Genes Dev.

[14] Irwin MS, Park JR (2015). Neuroblastoma: paradigm for precision medicine. Pediatr Clin North Am.

[15] Goldsmith KC, Gross M, Peirce S, Luyindula D, Liu X, Vu A, Sliozberg M, Guo R, Zhao H, Reynolds CP, Hogarty MD (2012). Mitochondrial Bcl-2 family dynamics define therapy response and resistance in neuroblastoma. Cancer Res.

[16] Lamers F, Schild L, den Hartog IJ, Ebus ME, Westerhout EM, Ora I, Koster J, Versteeg R, Caron HN, Molenaar JJ (2012). Targeted BCL2 inhibition effectively inhibits neuroblastoma tumour growth. Eur J Cancer.

[17] Souers AJ, Leverson JD, Boghaert ER, Ackler SL, Catron ND, Chen J, Dayton BD, Ding H, Enschede SH, Fairbrother WJ, Huang DC, Hymowitz SG, Jin S, Khaw SL, Kovar PJ, Lam LT (2013). ABT-199, a potent and selective BCL-2 inhibitor, achieves antitumor activity while sparing platelets. Nat Med.

[18] Mason KD, Carpinelli MR, Fletcher JI, Collinge JE, Hilton AA, Ellis S, Kelly PN, Ekert PG, Metcalf D, Roberts AW, Huang DC, Kile BT (2007). Programmed anuclear cell death delimits platelet life span. Cell.

[19] Roberts AW, Seymour JF, Brown JR, Wierda WG, Kipps TJ, Khaw SL, Carney DA, He SZ, Huang DC, Xiong H, Cui Y, Busman TA, McKeegan EM, Krivoshik AP, Enschede SH, Humerickhouse R (2011). Substantial susceptibility of chronic lymphocytic leukemia to BCL2 inhibition: results of a phase I study of navitoclax in patients with relapsed or refractory disease. J Clin Oncol.

[20] Rudin CM, Hann CL, Garon EB, Ribeiro de Oliveira M, Bonomi PD, Camidge DR, Chu Q, Giaccone G, Khaira D, Ramalingam SS, Ranson MR, Dive C, McKeegan EM, Chyla BJ, Dowell BL, Chakravartty A (2012). Phase II study of single-agent navitoclax (ABT-263) and biomarker correlates in patients with relapsed small cell lung cancer. Clin Cancer Res.

[21] Wilson WH, O'Connor OA, Czuczman MS, LaCasce AS, Gerecitano JF, Leonard JP, Tulpule A, Dunleavy K, Xiong H, Chiu YL, Cui Y, Busman T, Elmore SW, Rosenberg SH, Krivoshik AP, Enschede SH (2010). Navitoclax, a targeted high-affinity inhibitor of BCL-2, in lymphoid malignancies: a phase 1 dose-escalation study of safety, pharmacokinetics, pharmacodynamics, and antitumour activity. Lancet Oncol.

[22] Zhang H, Nimmer PM, Tahir SK, Chen J, Fryer RM, Hahn KR, Iciek LA, Morgan SJ, Nasarre MC, Nelson R, Preusser LC, Reinhart GA, Smith ML, Rosenberg SH, Elmore SW, Tse C (2007). Bcl-2 family proteins are essential for platelet survival. Cell Death Differ.

[23] Pan R, Hogdal LJ, Benito JM, Bucci D, Han L, Borthakur G, Cortes J, DeAngelo DJ, Debose L, Mu H, Dohner H, Gaidzik VI, Galinsky I, Golfman LS, Haferlach T, Harutyunyan KG (2014). Selective BCL-2 inhibition by ABT-199 causes on-target cell death in acute myeloid leukemia. Cancer Discov.

[24] Vandenberg CJ, Cory S (2013). ABT-199, a new Bcl-2-specific BH3 mimetic, has *in vivo* efficacy against aggressive Myc-driven mouse lymphomas without provoking thrombocytopenia. Blood.

[25] Choo EF, Boggs J, Zhu C, Lubach JW, Catron ND, Jenkins G, Souers AJ, Voorman R (2013). The role of lymphatic transport on the systemic bioavailability of the Bcl-2 protein family inhibitors navitoclax (ABT-263) and ABT-199. Drug Metab Dispos.

[26] Davids MS, Letai A (2013). ABT-199:taking dead aim at BCL-2. Cancer Cell.

[27] Touzeau C, Dousset C, Le Gouill S, Sampath D, Leverson JD, Souers AJ, Maiga S, Bene MC, Moreau P, Pellat-Deceunynck C, Amiot M (2014). The Bcl-2 specific BH3 mimetic ABT-199:a promising targeted therapy for t(11;14) multiple myeloma. Leukemia.

[28] Chen S, Dai Y, Harada H, Dent P, Grant S (2007). Mcl-1 down-regulation potentiates ABT-737 lethality by cooperatively inducing Bak activation and Bax translocation. Cancer Res.

[29] Choudhary GS, Al-Harbi S, Mazumder S, Hill BT, Smith MR, Bodo J, Hsi ED, Almasan A (2015). MCL-1 and BCL-xL-dependent resistance to the BCL-2 inhibitor ABT-199 can be overcome by preventing PI3K/AKT/mTOR activation in lymphoid malignancies. Cell Death Dis.

[30] Lestini BJ, Goldsmith KC, Fluchel MN, Liu X, Chen NL, Goyal B, Pawel BR, Hogarty MD (2009). Mcl1 downregulation sensitizes neuroblastoma to cytotoxic chemotherapy and small molecule Bcl2-family antagonists. Cancer Biol Ther.

[31] Mazumder S, Choudhary GS, Al-Harbi S, Almasan A (2012). Mcl-1 Phosphorylation defines ABT-737 resistance that can be overcome by increased NOXA expression in leukemic B cells. Cancer Res.

[32] Wang B, Ni Z, Dai X, Qin L, Li X, Xu L, Lian J, He F (2014). The Bcl-2/xL inhibitor ABT-263 increases the stability of Mcl-1 mRNA and protein in hepatocellular carcinoma cells. Mol Cancer.

[33] Czabotar PE, Lee EF, van Delft MF, Day CL, Smith BJ, Huang DC, Fairlie WD, Hinds MG, Colman PM (2007). Structural insights into the degradation of Mcl-1 induced by BH3 domains. Proc Natl Acad Sci U S A.

[34] Yecies D, Carlson NE, Deng J, Letai A (2010). Acquired resistance to ABT-737 in lymphoma cells that up-regulate MCL-1 and BFL-1. Blood.

[35] Gomez-Bougie P, Menoret E, Juin P, Dousset C, Pellat-Deceunynck C, Amiot M (2011). Noxa controls Mule-dependent Mcl-1 ubiquitination through the regulation of the Mcl-1/USP9X interaction. Biochem Biophys Res Commun.

[36] Gomez-Bougie P, Wuilleme-Toumi S, Menoret E, Trichet V, Robillard N, Philippe M, Bataille R, Amiot M (2007). Noxa up-regulation and Mcl-1 cleavage are associated to apoptosis induction by bortezomib in multiple myeloma. Cancer Res.

[37] Hauck P, Chao BH, Litz J, Krystal GW (2009). Alterations in the Noxa/Mcl-1 axis determine sensitivity of small cell lung cancer to the BH3 mimetic ABT-737. Mol Cancer Ther.

[38] Nakajima W, Hicks MA, Tanaka N, Krystal GW, Harada H (2014). Noxa determines localization and stability of MCL-1 and consequently ABT-737 sensitivity in small cell lung cancer. Cell Death Dis.

[39] Pang X, Zhang J, Lopez H, Wang Y, Li W, O'Neill KL, Evans JJ, George NM, Long J, Chen Y, Luo X (2014). The carboxyl-terminal tail of Noxa protein regulates the stability of Noxa and Mcl-1. J Biol Chem.

[40] Yan J, Zhong N, Liu G, Chen K, Liu X, Su L, Singhal S (2014). Usp9x- and Noxa-mediated Mcl-1 downregulation contributes to pemetrexed-induced apoptosis in human non-small-cell lung cancer cells. Cell Death Dis.

[41] Bate-Eya LT, Ebus ME, Koster J, den Hartog IJ, Zwijnenburg DA, Schild L, van der Ploeg I, Dolman ME, Caron HN, Versteeg R, Molenaar JJ (2014). Newly-derived neuroblastoma cell lines propagated in serum-free media recapitulate the genotype and phenotype of primary neuroblastoma tumours. Eur J Cancer.

[42] Dolman ME, Poon E, Ebus ME, den Hartog IJ, van Noesel CJ, Jamin Y, Hallsworth AM, Robinson SP, Petrie K, Sparidans RW, Kok RJ, Versteeg R, Caron HN, Chesler L, Molenaar JJ (2015). Cyclin-dependent kinase inhibitor AT7519 as a potential drug for MYCN-dependent neuroblastoma. Clin Cancer Res.

[43] Twentyman PR, Luscombe M (1987). A study of some variables in a tetrazolium dye (MTT) based assay for cell growth and chemosensitivity. Br J Cancer.

[44] Chou TC, Talalay P (1977). A simple generalized equation for the analysis of multiple inhibitions of Michaelis-Menten kinetic systems. J Biol Chem.

