# Supplementary material for: High efficacy of the BCL-2 inhibitor ABT199 (venetoclax) in BCL-2 high-expressing neuroblastoma cell lines and xenografts and rational for combination with MCL-1 inhibition
Source: Oncotarget. 2016 Apr 1;7(19):27946–58. doi: 10.18632/oncotarget.8547 (PMC5053701; doi:10.18632/oncotarget.8547)
Supplement: Supplementary file 1 [file oncotarget-07-27946-s001.pdf]

SUPPLEMENTARY MATERIALS AND METHODS

Cell culture

Classical human neuroblastoma cell lines were grown in Dulbecco's modified Eagle's medium (DMEM) containing 4.5 g/L D-glucose, glutamate and supplemented with 10% (v/v) foetal calf serum, 2 mmol/L L-glutamine, 10 U/mL penicillin, 10 µg/mL streptomycin and MEM non-essential amino acids (1x). Neuroblastoma tumour-initiating cell (TIC) lines were grown in neural specific stem cell medium (400 mL DMEM GlutaMAX™-1 containing 1g/L D-glucose and pyruvate, 133 mL F12 medium, 10 mL B27, 18 ng/mL EGF, 36 ng/mL FGF, 10 U/mL penicillin and 10 µg/mL streptomycin. Cells were maintained at 37°C under 5% CO_2 in humidified air. Penicillin and streptomycin were obtained from Sigma Aldrich, EGF from Corning Life Sciences and FGF from PeproTech. Other cell culture related materials were obtained from Life Technologies.

In vitro FACS analysis

High BCL-2-expressing neuroblastoma cell lines CHP126, KCNR and SJNB12 and low BCL-2-expressing cell lines SKNAS and SHEP2 were seeded in triplicate onto 6-cm plates and incubated overnight. Cells were then 72-hour treated with 0.1% DMSO (control) or ABT199 using concentration ranges of 7.8 nmol/L to 10 µmol/L. Supernatants containing floating cells were collected from the culture dishes. Adherent cells were washed once with PBS and PBS solutions were pooled with the supernatants. After trypsinization of the adherent cells with 0.05% trypsin/EDTA, cells were resuspended in the pooled supernatant/PBS solution. Next, cells were centrifuged (5 min; 1,500 rpm), washed by resuspension in PBS and centrifuged again (5 min; 3,000 rpm). Cells were fixed with 100% ice-cold ethanol, stained with 0.05 mg/mL propidium iodide and supplemented with 0.05 mg/mL RNAse A in PBS. After 1-hour incubation in the dark at room temperature (RT), cells were filtered through a 50 µm filter (BD Biosciences) and DNA contents of the nuclei were analyzed using a fluorescence-activated cell sorter. A total of 20,000 nuclei per sample were counted. The cell cycle distribution and apoptotic sub-G_1 fraction were determined using the BD Accuri™ C6 flow cytometer with the CFlow plus software (BD Biosciences).

Cell fractionation

CHP126, KCNR, SJNB12, SKNAS and SHEP2 were seeded onto 14-cm culture dishes and incubated overnight. Cell lines were then 24-hour treated with 0.04% DMSO (control) or 62.5 nmol/L to 10 µmol/L ABT199. Supernatants containing floating cells were collected from the culture dishes. Adherent cells were washed once with PBS and the PBS solutions were pooled with the supernatants. After trypsinization of the adherent cells with 0.05% trypsin/EDTA, cells were resuspended in the pooled supernatant/PBS solution. Next, cells were centrifuged (5 min; 1,500 rpm), twice washed by resuspension in PBS and centrifuged again (5 min; 1,500 rpm). Cytosolic and organelle fractions were subsequently separated using the ProteoExtract® Subcellular Proteome Extraction Kit according to the manufacturers protocol (Calbiochem, 539790). Cell fractions were used for cytochrome c detection by Western blot analysis (see below).

In vitro Western blotting

Cells were seeded onto 9- or 14-cm dishes and treated with DMSO (control) or up to 10 µmol/L ABT199. Treated cells were harvested at indicated time points and lysed using Laemmli buffer [i.e., H_2O/glycerol/20% sodium dodecyl sulfate (SDS)/1 M Tris-HCl (pH 6.8) 5:2:2:1 (v/v/v/v)]. Lysates were homogenized by hydrodynamic shearing through a 23 G needle, followed by 10 min incubation at 50ºC. Protein concentrations were determined using the Bio-Rad *DC* Protein Assay (Bio-Rad, Veenendaal, the Netherlands).

Equal protein amounts (40 µg) were diluted in 5x reducing sampling buffer (i.e., Laemmli buffer/β-mercaptoethanol 3:1 (v/v) with bromophenol blue sodium salt). Diluted samples were boiled for 5 min at 95°C and centrifuged (1,500 rpm 5 min). Proteins were separated by SDS-polyacrylamide gel electrophoresis on 12% Mini-Protean® Tris-glycince extended (TGX) precast gels (Bio-Rad) and transferred on hybond nitrocellulose membranes (0.45 µm) by 1.5-hour wet blotting (200 mA; 4ºC). Transfer buffer consisted of 20% (v/v) methanol, 3.025 g/L Tris and 14.4 g/L glycine in demineralized water. Membranes were blocked in 2% ECL Prime™ blocking agent (GE Healthcare) in PBS with 0.1% (v/v) Tween-20 (= blocking buffer) for 1 hour at RT. After blocking, membranes were incubated with the primary (overnight; 4ºC) and secondary (1 hour; RT) antibodies in blocking buffer and scanned using the Image Quant LAS 4000 detection system (GE Healthcare Life Science).

In vitro co-immunoprecipitation and immunoblotting

For all samples, total cell lysates were prepared in 2% Chaps buffer (i.e., 1 mol/L HEPES, 150 mmol/L NaCl, 5 mmol/L EDTA, 5% sodium glycerol phosphate (w/v) and 2% (w/v) Chaps). Equal protein amounts (1 mg) were added to antibody-matrix complex Protein A-Agarose

beads (Roche) for 24 hours at 4°C. Immunoprecipitated proteins were released from the matrix using RIPA buffer (1x) and analyzed by Western blot analysis as described above, with minor modifications. MCL-1 detection has been performed using mouse anti-human MCL-1 (clone RC13) monoconal antibody (Merck Millipore). Other antibodies were similar to the antibodies used for *In vitro* Western blotting and all antibodies have been used in a 1:100 dilution. Flag control samples were immunoprecipitated with an anti-Flag rabbit DYDDDDK tag antibody (clone 2368) (1:100 Cell Signaling Technology).

In vivo efficacy in neuroblastoma mouse models

Female NMRI *nu*⁻/*nu*⁻ mice (6-15 weeks old; 20-30 g) were subcutaneously injected with 1-5x10^6 cells/flank of KCNR. The size of the tumors was recorded twice weekly and when the tumors reached a size of approximately 1,000 mm^3, tumor pieces were serially xenotransplanted in recipient mice. Formalin-fixed and paraffin-embedded sections of the serially xenotransplanted tumors were routinely checked by hematoxylin-eosin staining. For the efficacy studies, recipient mice with KCNR neuroblastoma xenografts of approximately 268 mm^3 were orally treated with 100 mg/kg/d ABT199 (n = 5), 100 mg/kg/d ABT263 (n = 5), or vehicle (n = 6) for 21 days. Tumor sizes were measured by an external caliper.

In vivo immunohistochemistry

Paraffin-embedded sections of 4 µm were deparaffinized in xylene and hydrated in a series of alcohol baths. Endogenous peroxidase activity was inactivated by 10 minutes incubation with 0.3% (v/v) hydrogen peroxide in methanol. After rinsing in running tap water (5 min), tumor sections were pre-treated with 10/1 mmol/L Tris/EDTA buffer pH 9.1 (10 min; wet autoclaving). Sections were rinsed in Tris buffered saline (TBS) pH 7.8 (5 min) and 1-hour incubated with the primary antibodies. Anti-Ki67- and anti-cleaved caspase-3 antibodies were diluted in ScyTek Antibody Diluent (ScyTek Laboratories). Sections were washed with TBS (3x 2 min), incubated with the secondary antibody, rinsed again in TBS and incubated using the 3,3-diaminobenzidine (DAB) + Substrate Chromogen System (10 min; Dako, Heverlee, Belgium). Nuclei were stained by successively rinsing with running tap water, counterstaining with hematoxylin and rinsing again with running tap water. Sections were dehydrated in an ascending graded series of ethanol baths and xylene and mounted with Pertex and coverslip. Prior to visualization with an Olympus BX51 Microscope and DP70 Digital Camera System, sections were overnight incubated at 50ºC. All incubations were performed at RT, unless indicated otherwise.

RNA isolation and real-time quantitative PCR

RNA was isolated using the Trizol-chloroform method from the KCNR cell line treated for 24-hour with 32.5-125 nmol/L concentrations of ABT199. 1 µg of mRNA was reversed transcribed using the Taqman reverse transcription kit and amplified using the SYBR Green Master Mix (Applied Biosystems, N808234) and examined on a MyiQ Real-time PCR systems (Bio-Rad). Quantitative, real-time PCR was performed using the following specific primers: *MCL-1* 5’-ATGCTTCGGAAACTGGACAT-3’(forward) and 5’-TCCTGATGCCACCTTCTAGG-3’(reverse); *Noxa* 5’-TGGAAGTCGAGTGTGCTACTCAA-3’ (forward) and 5’-CAGAAGA GTTTGGATATCAGA TTCAGA-3’ (reverse) and *B-ACTIN* 5’-AGAAAATCTGG CACCACACC-3’ (forward) and 5’-AGAGGCGTACA GGGATAGCA-3’ (reverse).

SUPPLEMENTARY FIGURES AND TABLE

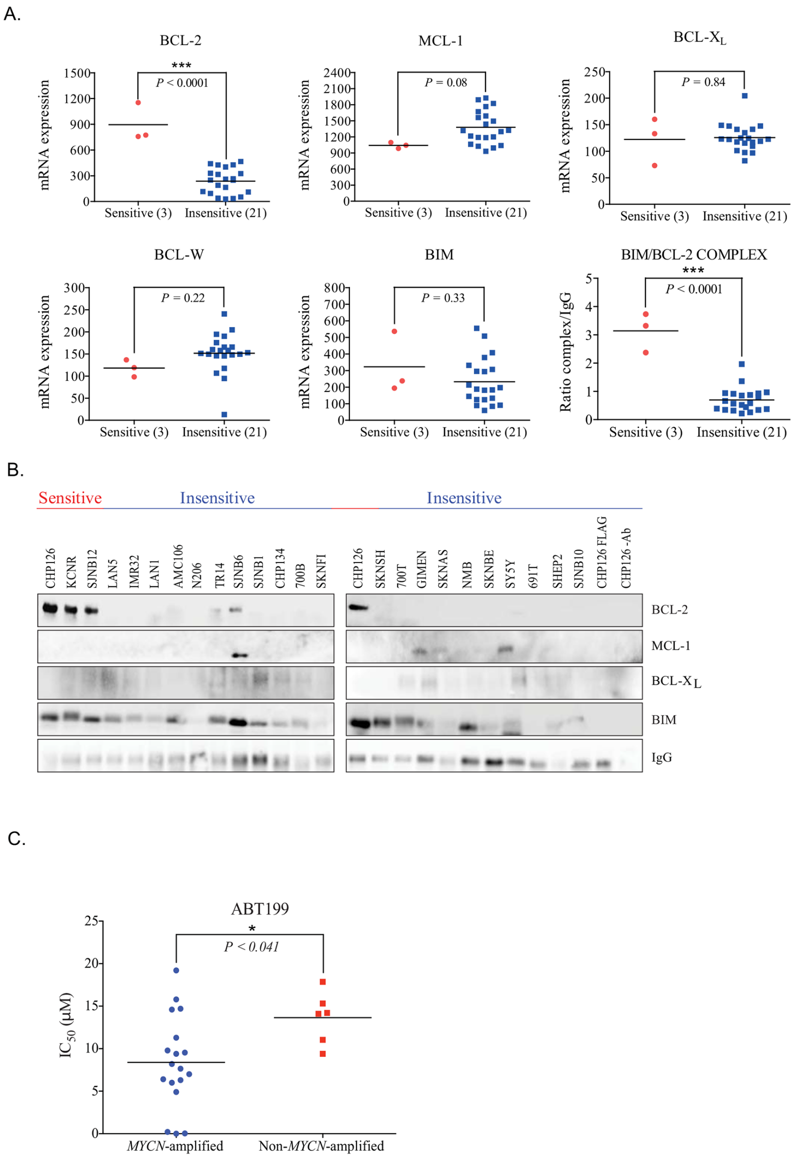

Supplementary Figure S1: *BCL-2* mRNA levels, protein levels of BCL-2/BIM complex and MYCN amplification status predict sensitivity to ABT199. A. mRNA expression levels of *BCL-2*, *MCL-1*, *BCL-X$_L$*, *BCL-W* and *BIM* and BCL-2/BIM protein complex levels in sensitive (i.e., CHP126, KCNR and SJNB12) versus insensitive neuroblastoma cell lines. *BCL-2*, *MCL-1*, *BCL-X$_L$*, *BCL-W* and *BIM* mRNA levels have been established using the R2 bioinformatics platform (http://r2.amc.nl), which contains Affymetrix mRNA expression data of all cell lines studied in the current manuscript. BCL-2/BIM complex levels were established by anti-BIM immunoprecipitation of whole cell lysates, followed by Western blotting for BCL-2. BCL-2 band intensities were normalized to the IgG heavy chain of the BIM antibody. Statistical differences between the sensitive and insensitive cell lines were calculated using a one-tailed (for *BCL-2* mRNA and BIM/BCL-2 protein complex) or two-tailed unpaired Student *t* test, with $P < 0.05$ as the minimal level of significance and $P < 0.0001$ indicated as ***. Horizontal lines represent the mean of the mRNA expression of the BCL-2 family proteins and the BCL-2/BIM complex levels of the cell line panel. **B.** BCL-2/BIM, MCL-1/BIM and BCL-X$_L$/BIM complex levels in 24 neuroblastoma cell lines, ordered from ABT199 sensitive (left) to ABT199 insensitive (right). Complex levels were established by anti-BIM immunoprecipitation of whole cell lysates, followed by BCL-2, MCL-1 and BCL-X$_L$. The IgG heavy chain of the BIM antibody served as a loading control. Total protein levels of the BCL-2-like family member proteins of the cell line panel in Figure 1A served as whole cell lysates for this experiment. **C.** IC$_{50}$ values of MYCN amplified versus non-MYCN amplified cell lines treated with ABT199. Cell viability curves were obtained by MTT assays after 72-hour treatment of the cell lines with ABT199 and IC$_{50}$ values were calculated. Statistical differences in sensitivity to ABT199 between the MYCN and non-MYCN amplified cell lines were calculated using a one-tailed Student *t* test, with $P < 0.05$ as the minimal level of significance and $P < 0.0001$ indicated as ***.

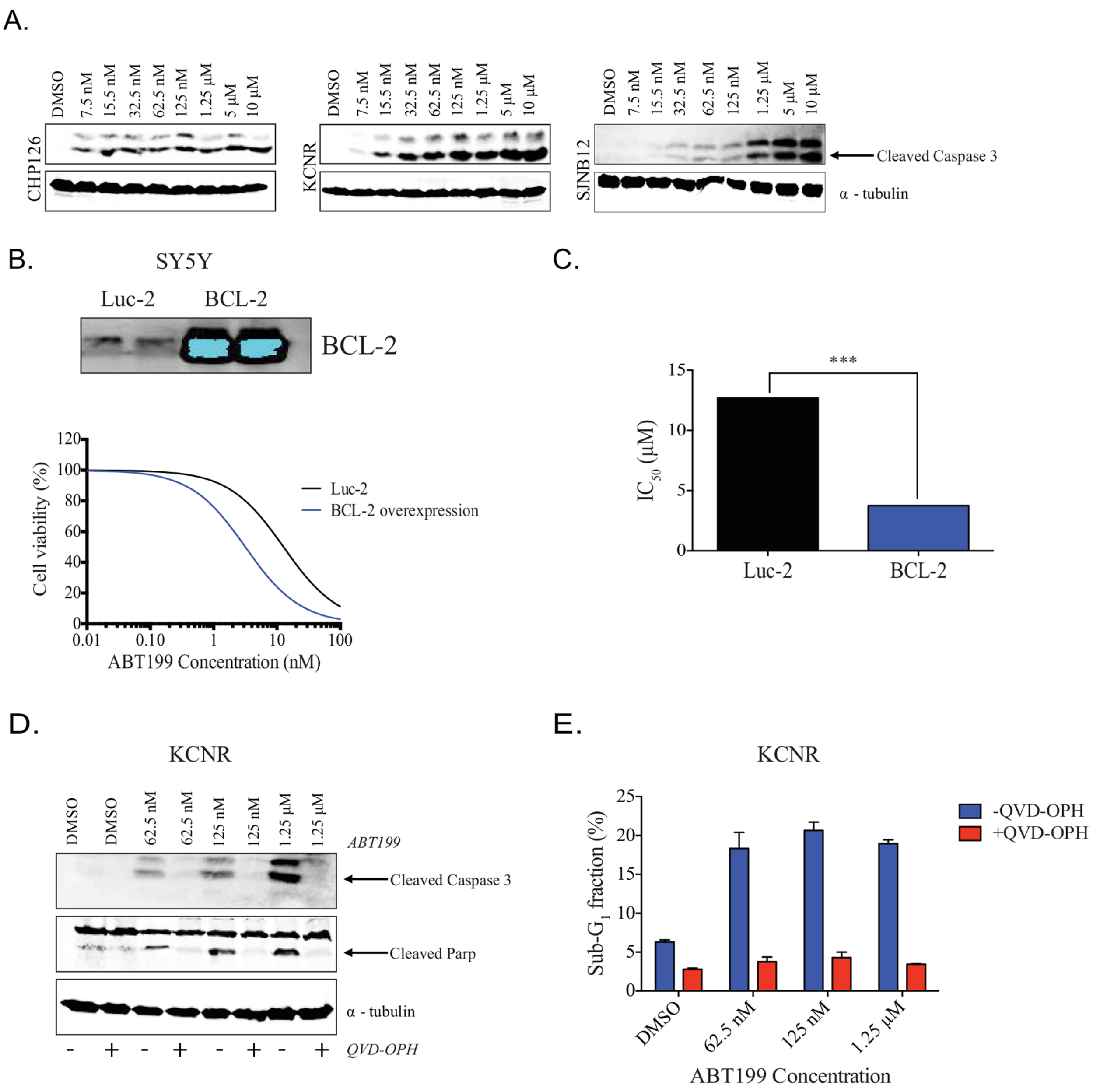

Supplementary Figure S2: ABT199 inhibits BCL-2 and causes apoptosis by induction of the intrinsic apoptotic pathway which can be rescued with caspase inhibitors. A. Western blot analysis of the *in vitro* effects of ABT199 on caspase 3 cleavage after 72-hour treatment of sensitive cell lines CHP126, KCNR and SJNB12 with increasing ABT199 concentrations. Alpha-tubulin served as loading control. **B.** The upper figure shows the Western blot analysis of the BCL-2 expression in SY5Y neuroblastoma cells transfected with either a luciferase 2 construct (i.e, control cells) or a pLenti 6/V5-DEST vector constitutively overexpressing the BCL-2 protein. Dose-response curves below show the difference in ABT199 sensitivity between the BCL-2-overexpressing and control SY5Y cells after 72-hour treatment. Curves were fitted based on a sigmoidal dose-response model using GraphPad Prism® software. ABT199 was tested in 10 concentrations and all concentrations were measured in triplicate. **C.** Bar graphs comparing the ABT199 IC_{50} values between SY5Y cells expressing the luciferase 2 construct and SY5Y overexpressing BCL-2. Data represent 1 experiment. **D.** Western blot analysis of the *in vitro* effects of the inhibition of caspase 3 and PARP cleavage after 4-hour treatment with the pan-caspase 3 inhibitor QVD-OPH and 24-hour treatment with the BCL-2 inhibitor ABT199 in the BCL-2 high expressing cell line KCNR. Alpha-tubulin served as loading control. **E.** FACS analysis of the *in vitro* effects on sub-G_1 induction after 4-hour treatment of KCNR with the pan-caspase inhibitor QVD-OPH and 24-hour treatment with increasing ABT199 concentrations. Data represent the mean percentages of cells in sub-G_1 ± SD of three replicate experiments.

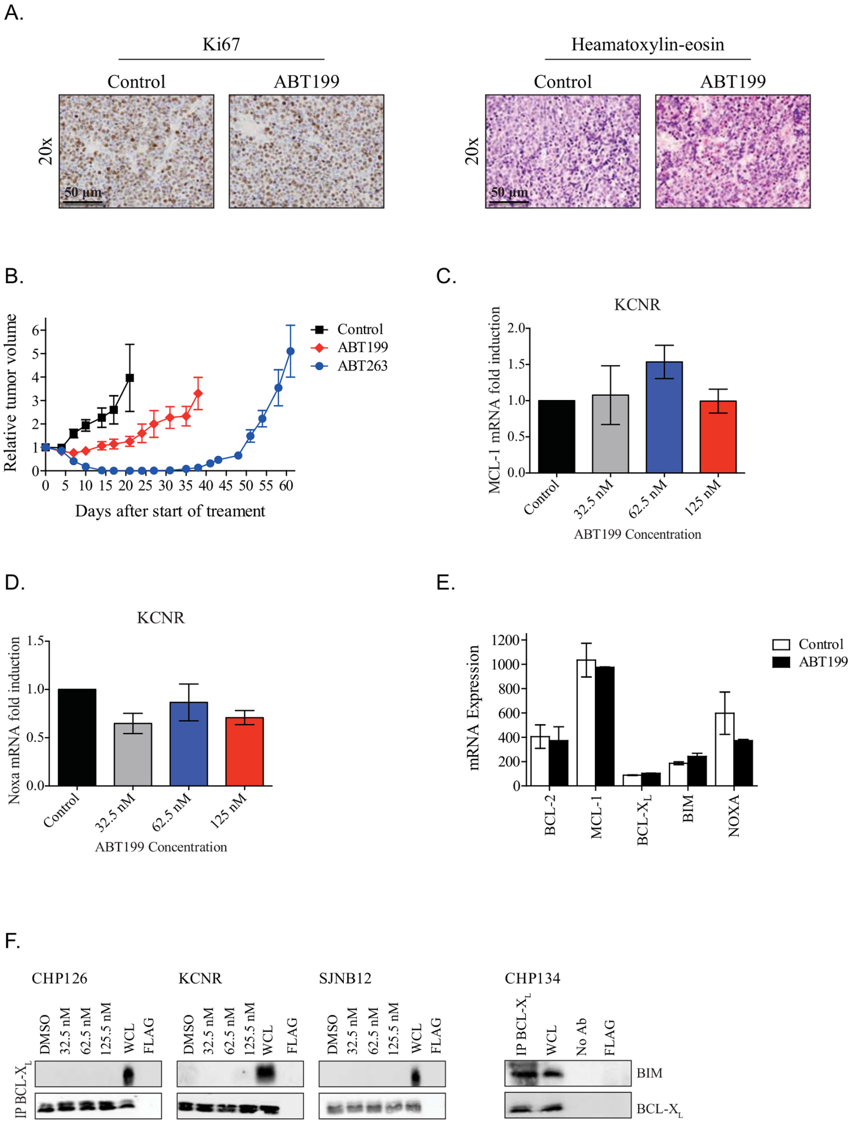

Supplementary Figure S3: ABT199 and ABT263 elicit anticancer activity in mice with KNCR neuroblastoma xenografts expressing high BCL-2 and BIM/BCL-2 complex levels. Mice with KCNR xenografts of approximately 268 mm^3 were daily treated with vehicle (control), 100 mg/kg ABT199 or 100 mg/kg ABT263 for 3 consecutive weeks. **A.** representative microscopic images of Ki67 and heamatoxylin-eosin stained paraffin-embedded sections of control tumors versus tumors treated with ABT199. Tumor materials were collected at 4 hours after administration of the last dose. ABT199 effects on cell proliferation and phenotypic changes caused by ABT199 were studied by immunohistochemistry analysis of Ki67 and conventional heamatoxylin-eosin staining, respectively. Magnification of the images: 20x. Scale bar: 50 µm. **B.** long-term effects of ABT199 and ABT263 on the growth of KCNR neuroblastoma xenografts after three weeks treatment. Relative tumor volume was calculated as the volume at the indicated day after start of treatment divided by the volume prior to treatment initiation. Data represent the mean relative tumour volume ± SEM. [Group sizes at the start of treatment: n = 10 (control), n = 5 (ABT199), and n = 5 (ABT263)]. **C, D.** Effects on *In vitro* mRNA levels of *MCL-1* and *NOXA* in the BCL-2 high expressing cell line KCNR treated for 24-hour with increasing concentrations of ABT199. *MCL-1* and *NOXA* fold change was analyzed by Real-time quantitative-PCR. RNA levels of the control treated samples were set to 1 for analysis. Data represent average mRNA expression values ± SD. **E.** ABT199 effects on *In vivo* mRNA levels of *BCL-2*, *MCL-1*, *BCL-X$_L$*, *BIM* and *NOXA*. Tumor materials of control and ABT199-treated KCNR xenografts (n = 2 per group) were collected at 4 hours after administration of the last dose and analyzed by Affymetrix mRNA profiling. Data represent average mRNA expression values ± SEM. **F.** *In vitro* effects of ABT199 on BIM/BCL-X_L complex levels in CHP126, SJNB12 and KCNR after 24-hour treatment with increasing doses. BIM/BCL-X_L complex levels were established by immunoprecipitation of BCL-X_L followed by Western blot analysis of BIM. Immunoprecipitated levels of BCL-X_L were used as loading controls. As no BIM/BCL-X_L complex could be detected in the cell lines tested, neuroblastoma cell line CHP134 was included as a positive control for BIM/BCL-X_L complex.

Supplementry Table S1: ***In vitro*** **effects on sub-G1 induction and cell cycle progression in** ***BCL-2*** **high expressing versus** ***BCL-2*** **low expressing neuroblastoma cell lines after 72 h treatment with ABT199**

		Sub-G1 (%)	G1 (%)	S (%)	G2 (%)
**CHP126**	0 nM	4.3±1.1	58.9±3.6	19.6±2.2	19.4±3.8
	7.5 nM	12.2±1.2	58.1±5.3	13.6±2.7	14.6±1.6
	15.5 nM	11.9±1.6	57.3±3.1	14.5±1.6	17.6±1.4
	32.5 nM	11.3±2.8	59.8±2.1	14.1±0.4	15.1±1.1
	62.5 nM	15.6±2.3	56.6±2.1	13.8±2.4	15.6±1.9
	125 nM	33.9±1.1	51.1±5.6	9.5±5.4	7.2±3.6
	1.25 µM	34.5±0.9	46.3±7.1	9.8±3.4	9.1±2.7
	5 µM	35.7±9.8	45.2±7.5	9.9±0.9	9.3±1.9
	10 µM	48.3±3.1	39.3±1.4	7.3±0.9	6.6±0.7
**KCNR**	0 nM	1.1± 0.5	58.69±0.9	21.53±0.8	18.26±0.9
	7.5 nM	14.4±1.6	55.05±2.9	19.39±1.9	11.35±2.4
	15.5 nM	17.3±3.0	49.89±0.5	21.22±0.02	12.05±3.5
	32.5 nM	22.5±2.7	47.73±1.6	18.65±1.6	10.59±1.5
	62.5 nM	22.0±2.9	49.65±1.1	17.75±0.3	9.84±0.04
	125 nM	20.2±1.6	53.91±2.2	17.02±3.4	9.40±0.5
	1.25 µM	21.1±1.3	52.50±6.4	17.15±4.1	10.54±3.3
	5 µM	24.9±0.4	48.45±0.6	16.45±1.2	9.21±0.3
	10 µM	26.3±1.8	46.13±0.8	17.62±0.5	9.81±0.1
**SJBB12**	0 nM	2.01±0.01	70.3±0.6	8.1±5.9	19.2±8.8
	7.5 nM	7.15±1.2	63.5±2.1	5±0.4	24.3±0.5
	15.5 nM	15.3±1.5	57.4±0.9	5.3±1.6	21.7±0.6
	32.5 nM	18±1.1	57.2±1.7	4.6±1.9	20±2.8
	62.5 nM	21.67±3.6	54.75±3.8	6.7±1.1	17.2±0.3
	125 nM	25.12±4.4	52.4±4.8	6.6±0.9	15.9±1.2
	1.25 µM	28.76±3.3	49.3±4.6	7±1.2	15.6±1.3
	5 µM	29.07±2.5	48.9±2.7	7.5±0.7	15.7±2.1
	10 µM	39.1±2.3	45.1±1.2	4.6±1.7	11.3±1.7
**SKNAS**	0 nM	1.3±0.6	59.4±6.3	17.9±1.6	21.4±4.3
	7.5 nM	1.8±1.2	58.2±2.1	18.9±0.5	19.3±1.7
	15.5 nM	1.7±0.7	58.3±4.1	19.9±1.1	20.9±4.6
	32.5 nM	1.1±0.4	59.1±3.7	19.1±0.3	20.1±3.1
	62.5 nM	1.3±0.2	60.1±3.3	19.6±0.2	19.3±3.4
	125 nM	1.2±0.1	59.6±1.1	20.1±1.1	18.4±1.6
	1.25 µM	1.3±0.2	58.7±1.2	19.5±1.2	18.7±2.6
	5 µM	1.4±0.09	58.8±2.6	21.6±2.6	17.4±0.6
	10 µM	8.1±5.6	59.7±4.9	15.3±4.9	16.5±4.7
**SHEP2**	0 nM	0.8±0.4	75.6±2.1	13.7±2.4	10.7±3.6
	7.5 nM	1.6±1.1	74.6±0.9	11.7±0.9	12.1±2.9
	15.5 nM	1.7±1.2	76.1±3.7	12.01±0.8	12.1±3.2
	32.5 nM	1.3±0.3	75.7±2.3	11.3±1.3	12.6±1.3
	62.5 nM	1.4±0.4	77.1±1.5	10.4±1.2	11.6±1.8
	125 nM	1.4±1.1	77.9±2.6	11.0±1.6	10.4±3.3
	1.25 µM	1.9±1.8	75.4±2.9	10.3±1.9	11.9±3.6
	5 µM	3.6±2.4	72.1±2.8	12.8±0.4	12.1±2.3
	10 µM	4.3±2.2	70.8±4.4	12.6±1.1	11.1±3.9

Values represent the average percentage of cells in sub-G1, G1, S and G2 +/− SD.
